# Supplementary material for: Improved economic and clinical outcomes with oritavancin versus a comparator group for treatment of acute bacterial skin and skin structure infections in a community hospital
Source: PLoS One. 2021 Mar 18;16(3):e0248129. doi: 10.1371/journal.pone.0248129 (PMC7971560; doi:10.1371/journal.pone.0248129)
Supplement: Attachment — Submitted filename: minor modifications.pdf [file pone.0248129.s001.pdf]

Dear Editor，

Thank you for your invitation. Generally accepted this paper, except for minor modifications.

Oritavancin is a lipoglycopeptide antibiotic. The US Food and Drug Administration (FDA) approved oritavancin in 2014 for adults with acute bacterial skin and skin structure infections (ABSSSI). In the current healthcare environment, there is substantial pressure to reduce costs and resource use while maintaining or improving quality of care. Oritavancin demonstrates concentration-dependent bactericidal activity and has a half-life of 245 hours. This half-life allows for treatment of ABSSSI with a single 1,200 mg IV dose, which has been shown to be non-inferior to vancomycin dosed twice daily for 7 to 10 days. Some study showed that oritavancin was clinically comparable to other antibiotics, but less costly and resource intensive.

This retrospective study compared the hospital experience and cost of a ABSSI patient with oritavancin and other antibiotics at a community hospital. From the text, oritavancin seems to have a slight advantage. Here are some places need to be thought about or answer：

1. Is it possible to expand the scope: in the case of the same other conditions, how direct prescription of oritavancin improves economic and clinical outcomes compared with other antibiotics.
2. Line 202, lack of word “aHC”, following “average”.

3. Line 381,table1. better add two columns with “ total”and “P value” .

4. Line 381,table1. BMI category, “>25” indented inward.

5. Line 381, table 1. better add line “prognosis， such as cured, readmission”.

6. Line 383,table 2, better add “medication costs avoidance” and “non-medication related costs avoidance” column.

7. Since not all patients are readmitted to the hospital,the costs avoidance of cured patients need to be compared. Add in table 2.

8. 90 days are long, how to approve the “recurrence” or “lack of improvement due to previous etiology”? the same body part? Or the same microbial? Or the second infection?

That is all ,thank you .

Dr. CHEN
