# Supplementary material for: Improved economic and clinical outcomes with oritavancin versus a comparator group for treatment of acute bacterial skin and skin structure infections in a community hospital
Source: PLoS One. 2021 Mar 18;16(3):e0248129. doi: 10.1371/journal.pone.0248129 (PMC7971560; doi:10.1371/journal.pone.0248129)
Supplement: Attachment — Submitted filename: Response to Reviewers (1).docx [file pone.0248129.s002.docx]

# Response to Reviewers

Thank you all for your time, attention, and invitation for submitting a revised submission of our manuscript. The authors’ responses are italicized below.

1. Please ensure that your manuscript meets PLOS ONE's style requirements, including those for file naming. The PLOS ONE style templates can be found at

https://journals.plos.org/ plosone/s/file?id=wjVg/ PLOSOne_formatting_sample_ main_body.pdf and

https://journals.plos.org/ plosone/s/file?id=ba62/ PLOSOne_formatting_sample_ title_authors_affiliations.pdf

*We have reviewed the above mentioned templates and made appropriate edits to meet the PLOS ONE’s style requirements.*

2. In the ethics statement in the manuscript and in the online submission form, please provide additional information about the patient records used in your retrospective study, including: a) whether all data were fully anonymized before you accessed them; b) the date range (month and year) during which patients' medical records were accessed; c) the date range (month and year) during which patients whose medical records were selected for this study sought treatment. If the ethics committee waived the need for informed consent, or patients provided informed written consent to have data from their medical records used in research, please include this information.

*The following has been added to the Methods section starting at line 150:*

*This study was approved by DeTar Healthcare System Ethics Committee and Chief Compliance Officer. Informed consents were waived by the Ethics Committee as Protected Health Information was not included and data was collected retrospectively. Patient data was collected via electronic medical records in May of 2019 for patients treated during the time period of August 2015 thru December of 2018. Patient data was anonymized following completion of data collection. This case study was performed in accordance with the Helsinki Declaration of 1964 and its later amendments. Waivers were approved by the hospital IRB.*

3. Your ethics statement should only appear in the Methods section of your manuscript. If your ethics statement is written in any section besides the Methods, please delete it from any other section.

*The ethics statement has been removed from the Acknowledgments section and provided in the Methods section.*

4.We note that you have indicated that data from this study are available upon request. PLOS only allows data to be available upon request if there are legal or ethical restrictions on sharing data publicly. For information on unacceptable data access restrictions, please see http://journals.plos.org/ plosone/s/data-availability# loc-unacceptable-data-access- restrictions.

In your revised cover letter, please address the following prompts:

a) If there are ethical or legal restrictions on sharing a de-identified data set, please explain them in detail (e.g., data contain potentially identifying or sensitive patient information) and who has imposed them (e.g., an ethics committee). Please also provide contact information for a data access committee, ethics committee, or other institutional body to which data requests may be sent.

*The cover letter has been revised to include restrictions on release of patient data set as imposed by the DeTar Ethics Committee and Compliance Officer.*

5.Thank you for stating the following in the Acknowledgments Section of your manuscript:

"The fees associated with publication of this study, including Rapid Service Fee

and Open Access fee, has been provided by Dr. Miguel Sierra-Hoffman, DeTar

Healthcare System, Victoria Emergency Associates, and the DeTar Family Medicine

Residency Program."

We note that you have provided funding information that is not currently declared in your Funding Statement. However, funding information should not appear in the Acknowledgments section or other areas of your manuscript. We will only publish funding information present in the Funding Statement section of the online submission form.

Please remove any funding-related text from the manuscript and let us know how you would like to update your Funding Statement. Currently, your Funding Statement reads as follows:

"The authors received no specific funding for this work."

*The authors received no specific funding for this work. The above statement found in the Acknowledgments section has been removed. The only funding to be provided by the above mentioned entities is funding for publication.*

We note that one or more of the authors have an affiliation to the commercial funders of this research study : DeTar Healthcare System

a. Please provide an amended Funding Statement declaring this commercial affiliation, as well as a statement regarding the Role of Funders in your study. If the funding organization did not play a role in the study design, data collection and analysis, decision to publish, or preparation of the manuscript and only provided financial support in the form of authors' salaries and/or research materials, please review your statements relating to the author contributions, and ensure you have specifically and accurately indicated the role(s) that these authors had in your study. You can update author roles in the Author Contributions section of the online submission form.

Please also include the following statement within your amended Funding Statement.

“The funder provided support in the form of salaries for authors [insert relevant initials], but did not have any additional role in the study design, data collection and analysis, decision to publish, or preparation of the manuscript. The specific roles of these authors are articulated in the ‘author contributions’ section.”

If your commercial affiliation did play a role in your study, please state and explain this role within your updated Funding Statement.

*The following has been added to the revised cover letter:*

*DeTar Healthcare System provided support in the form of a salary for author KS but did not have any additional role in the study design, data collection and analysis, decision to publish, or preparation of manuscript.*

b. Please also provide an updated Competing Interests Statement declaring this commercial affiliation along with any other relevant declarations relating to employment, consultancy, patents, products in development, or marketed products, etc.

Within your Competing Interests Statement, please confirm that this commercial affiliation does not alter your adherence to all PLOS ONE policies on sharing data and materials by including the following statement: "This does not alter our adherence to PLOS ONE policies on sharing data and materials.” (as detailed online in our guide for authors http://journals.plos.org/ plosone/s/competing-interests) . If this adherence statement is not accurate and there are restrictions on sharing of data and/or materials, please state these. Please note that we cannot proceed with consideration of your article until this information has been declared.

Please include both an updated Funding Statement and Competing Interests Statement in your cover letter. We will change the online submission form on your behalf.

*The Funding Statement and Competing Interests Statement have been updated in the cover letter.*

Reviewer #1: In this work Authors analyzed the economic and clinical impact of a long-acting anti-gram positive antibacterial for the treatment of ABSSSIs.

To do this, they compared 79 patients received oritavancin who had failed previous outpatient antibiotic therapy (OPAT) with 28 patients receiving other antibiotics following OPAT failure and subsequent hospitalization.

This work is well performed and interesting to read. Attached there are several comments to improve the manuscript.

Introduction:

Lines 101 – 110: this is an interesting point. Authors should briefly describe the reason of this increase in healthcare costs of SSTIs despite the decrease of MRSA infections

*Thank you for your observation. The following has been added to the Introduction section, starting at line 95: Within our facility, the rate of MRSA has remained constant with approximately 50% of Staphylococcus aureus infections attributed to MRSA over the last 3 years. Although a decrease in the rate of occurrence is reported, the cost to treat these infections continues to rise as healthcare costs continue to increase broadly.*

Methods:

Authors use alternatively the acronyms ABSSSI and SSTI, however they refer to two different classification. I suggest defining better and clearly type of infections included in this study.

*We have changed all references to SSTI to ABSSSI throughout the manuscript as introduced in the Introduction. Thank you for pointing this out.*

Results:

In this study the costs associated to antibiotics were not evaluated. However, if possible, I suggest also to consider costs associated with comparator antibiotics.

*We discussed this point but we did not feel we would be able to accurately assess the cost outpatient antibiotic therapy (OPAT). Further, the study was designed to focus on cost benefit and effectiveness of oritavancin in preventing subsequent readmissions, of which, the cost would far exceed the cost of OPAT.*

Discussion:

Use of long acting antibiotics that lead to early discharge of patients allows to reduce antimicrobial pressure in hospital setting. Reducing causes of antibiotic resistance is another important benefit of long-acting antibiotics. In my opinion this point and the need of including competencies in antibiotic use in all medical specialties should be briefly discussed (accordingly, this recent survey could be cited on this topic: “Italian young doctors' knowledge, attitudes and practices on antibiotic use and resistance: A national cross-sectional survey; J Glob Antimicrob Resist . 2020 Dec;23:167-173. doi: 10.1016/j.jgar.2020.08.022.”)

*This is an excellent point and we have built a robust AS program within our facility and again found our implementation of this protocol to prove success of interprofessional teamwork. We have added reference to the above article in the Discussion section. The following was added to the Discussion section starting at line 338: The combination of appropriate outpatient prescribing and the long acting effect of oritavancin may also contribute to decreased rates of antimicrobial resistance (AMR) by reducing antimicrobial pressure seen during hospitalization. As noted in a survey of young physicians by Gennero et al, it is widely recognized that AMR is a growing global issue but healthcare systems are lacking in addressing it at the local level [22]. In developing our local protocol, part of the aim was providing adequate education to providers on appropriate prescribing of oritavancin to promote antimicrobial stewardship (AS). Transitioning care to the outpatient setting reduces AMR well documented to occur with extended and frequent hospitalizations; while the long acting molecule affords assurance of completion of therapy, therefore, drastically reducing compliance as a cause for treatment failure.*

Reviewer #2: This article has some research value, but the scope of the topic is too large, or the scope of the research object is too small, it needs to be supplemented.The article only has the statistics data of hospitalized patients who had failed treatment.

*In our local healthcare system, we identified the positive outcomes we felt to be seen with the use of oritavancin and we aimed to validate our observations by performing this retrospective study. Our intent was to provide a real world experience of the benefit of appropriate prescribing of oritavancin and the resulting improvement in patient outcomes and decreased hospital costs resulting from reduced admissions.*

Below we address the comments and questions from Dr. Chen.

This retrospective study compared the hospital experience andcost of a ABSSI patient with oritavancin and other antibiotics at a communityhospital. From the text, oritavancin seems to have a slight advantage. Here are some places need to be thought about or answer：

1. Is it possible to expand the scope: in the case of the same other conditions, how direct prescription of oritavancin improves economicandclinical outcomes compared with other antibiotics.

*In our local healthcare system, we identified the positive outcomes we felt to be seen with the use of oritavancin and we aimed to validate our observations by performing this retrospective study. Our intent was to provide a real world experience of the benefit of appropriate prescribing of oritavancin and the resulting improvement in patient outcomes and decreased hospital costs resulting from reduced admissions.*

2. Line 202, lack of word “aHC”, following “average”.

*We were unclear of the meaning of this comment but did verify that “aHC” was identified as “average hospital cost” throughout the manuscript.*

3. Line 381,table1. better add two columns with “ total”and “P value”.

*In Table 1, patient demographic data is presented and due to the sample size, p Values were not helpful, rather averages were presented as appropriate. The differences noted in demographic data is discussed in the Results section beginning at line 204.*

4. Line 381,table1. BMI category, “>25” indented inward.

*Thank you for pointing this out. We have corrected the indentation difference in Table 1.*

5. Line 381, table 1. better add line “prognosis，such as cured, readmission”.

*Table 1 was used to summarize patient demographic data and Table 2 summarizes outcomes data including readmission rates due to failed therapy. In Table 3 we provided details of the patients in the oritavancin cohort that did not demonstrate complete resolution upon follow up and the subsequent provided treatments or further diagnosis. We hope that this provides sufficient data to outcomes as related to the study design.*

6. Line 383,table 2, better add “medication costs avoidance” and “non-medica tion related costs avoidance” column.

*Our focus for this study was to compare total hospital costs and the total hospital savings that resulted from reducing average length of stay and decreasing the rate of readmissions. We found the daily bed costs far outweighed the costs of inpatient antibiotic treatment and, thus, this data was not differentiated. Thank you for the observation and we will definitely consider this point in the writing of future publications. The following has been added to the Discussion section, starting at line 367, with additional reference for support: As reported by Ektare et al, hospital bed cost and average length of stay were the predominant factors in driving the total direct hospital costs for treating ABSSSI patients. In their analysis, the ability to move treatment of these patients to the outpatient setting demonstrated a potential cost savings of $13,090 to $13,473 by avoiding inpatient admission.*

7. Since not all patients are readmitted to the hospital,the costs avoidanceof cured patients need to be compared. Add in table 2.

*We were unsure how to interpret the above comment/question. In the study design, inclusion criteria was specific to patients who had failed previous therapy and required readmission, as such, we did not collect data on patients that were not readmitted.*

8. 90 days are long, how to approve the “recurrence” or “lack of improvement due to previous etiology”? the same body part? Or the samemicrobial? Or the second infection?

*We agree that 90 days is an extended time period for review of resolution of infections related to cellulitis or abscess. The 90 day time point was more of an exploratory data point and as demonstrated by the results, there was not a significant difference between the 30 day and 90 day readmission rates. The readmissions reported were specifically reviewed for documentation of same site infection and notation of failed treatment or lack of improvement in relation to the previous infection as specified by the medical provider. We carefully reviewed readmission documentation to verify that readmissions were indeed related to the initial infection as identified and included in the study data.*
